# The role of activity engagement, social connection, and masculinity norms in men's shed outcomes for male mental health: a narrative review

**DOI:** 10.3389/fpubh.2026.1884564

**Published:** 2026-07-15

**Authors:** Jared Kyle Sieusahai

**Affiliations:** School of Public Health, University of Alberta, Edmonton, AB, Canada

**Keywords:** activity engagement, community-based intervention, help-seeking, male mental health, masculinity norms, men's sheds, social connection, social isolation

## Abstract

**Background/objectives:**

Men's Sheds are community-based, male-focused spaces that use shared practical activities to foster social connection and wellbeing, particularly among older and retired men at risk of social isolation. Despite growing policy interest across Australia, Ireland, Scotland, the United Kingdom, and Canada, the mechanisms by which Men's Sheds produce mental health benefits remain poorly theorized and inconsistently measured. This narrative review synthesizes peer-reviewed evidence on how three intersecting mechanisms activity engagement, social connection, and masculinity norms shape mental health outcomes in Men's Sheds, with attention to Canadian and international contexts.

**Methods:**

A systematic search of PubMed was conducted in May 2026 using Boolean search strings targeting Men's Shed literature combined with psychosocial outcome and mechanistic terms, yielding 44 combined results. After screening against inclusion and exclusion criteria, 20 core Men's Shed studies were included, supplemented by contextual literature on male social isolation, masculinity norms, and Canadian men's mental health. The review was conducted in accordance with SANRA guidelines for narrative reviews.

**Results:**

Evidence from mixed-methods, qualitative, and emerging quantitative studies supports associations between Shed participation and reduced social isolation, improved mood, and enhanced wellbeing, particularly for men aged 50 and over. The most methodologically robust evidence comes from a path-model survey demonstrating that psychological safety predicts engagement, engagement predicts expanded social networks and meaning in life, and these in turn predict higher wellbeing and lower loneliness. Masculinity norms function as both a barrier to formal help-seeking and a facilitator of Shed engagement through alignment with masculine-coded activities and the ‘health by stealth' model. Significant evidence gaps persist around Indigenous, racialized, immigrant, 2SLGBTQ+, and working-age men, and around direct empirical measurement of masculinity norm change over time.

**Conclusions:**

Men's Sheds are a promising, context-sensitive complement to formal mental health services. Future research should employ longitudinal designs, standardized outcome measures including the UCLA-3, WEMWBS, PHQ-9, GAD-7, and CMNI-46, and apply established theoretical frameworks including Connell's hegemonic masculinity model and Addis and Mahalik's help-seeking framework to clarify how Sheds reproduce, renegotiate, or recompose masculine norms across diverse populations.

## 1 Introduction

Men experience substantially elevated rates of social isolation, loneliness, and undertreated mental health problems across the life course (1–4). Large epidemiological surveys indicate that loneliness is more prevalent in men than women and highest among younger men in individualistic cultures (5), while men remain more socially isolated than women at most ages, particularly when never married or with disrupted partnership histories (1). Among older adults, approximately one in five experience chronic loneliness, which independently predicts higher depression scores (2). Canadian data show that living alone and loneliness are associated with poorer positive mental health, especially reduced community belonging in older men (4, 6). At the end of life, approximately 19% of older adults are socially isolated and 18% are lonely in the last 4 years of life, with isolation rising nearer death (4). These patterns position older, retired, and relationship-disrupted men as a priority population for community-based mental health intervention.

Despite this burden, men systematically underutilize formal mental health services. A systematic review of 47 studies reports that traditional masculinity norms centered on strength, self-reliance, and emotional stoicism significantly deter men from seeking mental health support through both direct impacts on mental health and multiple barriers to help-seeking (7). These findings are theoretically grounded in Connell's concept of hegemonic masculinity (8), which describes how dominant masculine ideals are reproduced through social practice and create structural barriers to health-seeking behavior. Addis and Mahalik's (9) framework of masculine role conflict provides a complementary lens, explaining how men experience tension between their need for help and the perceived incompatibility of help-seeking with masculine identity. This framework predicts that interventions aligning with masculine norms such as activity-based community programs will generate less role conflict and lower barriers to engagement than clinic-based services. Qualitative evidence from Canada supports this picture: over half of employed men in a Canadian workplace survey reported keeping feelings to themselves, and almost half preferred not to discuss their problems, while a photovoice study found that peer support requests were often implicit and constrained by normative masculine identities (10, 11).

Men's Sheds have emerged as a gendered, community-based response to this convergence of problems. Originating in Australia and spreading to Ireland, Scotland, the United Kingdom, Canada, and beyond, Sheds bring men together for shared practical activity woodwork, metalwork, electronics, gardening and informal socializing (12, 13). National male health policies in Australia and Ireland explicitly identify Sheds as sites to address social isolation and promote health (12, 13), and a growing body of research has begun to examine how and why they work. This narrative review synthesizes that evidence with a specific focus on three intersecting mechanisms: activity engagement, social connection, and masculinity norms. In doing so, it extends prior narrative reviews of Men's Sheds (12, 13) by applying an explicit theoretical framework organized around these three mechanisms, integrating equity considerations for diverse male populations, and incorporating Canadian-specific evidence not addressed in earlier work.

## 2 Methods

This narrative review was conducted in accordance with the Scale for the Assessment of Narrative Review Articles (SANRA) guidelines (14). The review addresses the following aim: to synthesize peer-reviewed evidence on the mechanisms by which activity engagement, social connection, and masculinity norms shape mental health outcomes in Men's Sheds, with attention to Canadian and international contexts. This review extends prior narrative reviews (12, 13) by applying an explicit three-mechanism theoretical framework as its organizing structure, integrating equity considerations, and incorporating Canadian-specific evidence not addressed in earlier work.

### 2.1 Search strategy

A systematic literature search was conducted in PubMed on May 26, 2026. Three search strings were developed using Boolean operators to capture Men's Shed literature across two conceptual domains: psychosocial outcomes and mechanistic pathways. Search string 1 targeted Men's Shed literature specifically: “Men's Shed”[Title/Abstract] OR “Men's Sheds”[Title/Abstract] yielding 59 results. Search string 2 targeted psychosocial outcome terms: “mental health”[Title/Abstract] OR wellbeing[Title/Abstract] OR “social isolation”[Title/Abstract] OR loneliness[Title/Abstract] yielding 230,283 results. Search string 3 targeted mechanistic terms: masculinity[Title/Abstract] OR “help-seeking”[Title/Abstract] OR “social connection”[Title/Abstract] OR “activity engagement”[Title/Abstract] yielding 14,707 results. The primary combined search (Search 1 AND Search 2) yielded 44 results and was used as the basis for title and abstract screening. Search terms were selected to capture peer-reviewed literature in which Men's Sheds were a primary focus and to ensure coverage of the three mechanistic domains central to this review's theoretical framework. Supplementary searches were conducted in Google Scholar using targeted terms including ‘Men's Sheds masculinity,' ‘Men's Sheds Canada,' and ‘male help-seeking community intervention.' Reference lists of all included Men's Shed studies and systematic reviews were hand-searched to identify contextual literature.

### 2.2 Inclusion and exclusion criteria

Studies were included if they: (14) examined Men's Sheds as a primary focus or as a directly relevant community-based intervention for male mental health; (15) addressed mental health, wellbeing, social isolation, loneliness, masculinity norms, or help-seeking outcomes; (13) were published in English between 2000 and 2026; and (16) were peer-reviewed. Contextual literature on male social isolation, masculinity norms, and Canadian men's mental health was included where it directly informed the mechanistic framework. Studies were excluded if they focused on community programs not specifically oriented toward male health or wellbeing, or if they were conference abstracts, gray literature, or non-peer-reviewed reports.

### 2.3 Screening and selection

Titles and abstracts of the 44 results from the primary combined search were screened for relevance against the inclusion and exclusion criteria. Full texts of relevant studies were retrieved and assessed. Studies identified through supplementary searches and hand-searching were assessed using the same criteria. A total of 20 peer-reviewed sources directly addressing Men's Sheds were included as core evidence, supplemented by contextual literature on male social isolation, masculinity norms, and Canadian men's mental health, bringing the total included sources to 40.

### 2.4 Quality assessment

Given the narrative review format, formal risk-of-bias tools designed for systematic reviews were not applied. Methodological quality was assessed descriptively using SANRA criteria (14), with attention to study design, sample size, outcome measurement validity, and author-reported limitations. For qualitative studies, quality was assessed with reference to Critical Appraisal Skills Programme (CASP) criteria. For mixed-methods studies, Mixed Methods Appraisal Tool (MMAT) criteria were applied descriptively. Methodological strengths and limitations are discussed throughout the Results sections. Studies are not excluded on the basis of methodological quality alone; limitations are noted transparently to contextualize the strength of evidence presented.

## 3 Results

Table 1 presents a summary of the 20 core Men's Shed studies included in this review, organized by author, country, study design, sample, population, and key findings.

**Table 1:** Summary of included men's shed studies (NR = not reported).

References	Country	Study design	Sample	Population	Key findings
Wilson & Cordier (12)	Australia	Narrative review	24 studies	Older men in community Sheds	Foundational review; Sheds support informal learning, social wellbeing; most evidence descriptive; calls for standardized outcome measures and longitudinal designs
Cordier & Wilson (13)	Australia/Ireland	Cross-sectional survey	NR	Shed members, policy stakeholders	Sheds play dual health and social roles; embedded in national male health policy in Australia and Ireland as sites to address social isolation
Moylan et al. (35)	Australia	Qualitative/mixed	NR	Men's Shed members	Sheds provide biopsychosocial and spiritual support; social connection, sense of purpose, and belonging are central mechanisms
Southcombe et al. (25)	Australia	Qualitative	NR	Indigenous men in sheds/groups	Shed-based programs build capacity in Indigenous men's groups; culturally adapted approaches including community elder leadership essential for social and emotional wellbeing
Henwood et al. (27)	Australia	Mixed methods	NR	Men in community health organizations	Communities of practice in men's health organizations support social connection and peer learning; Sheds exemplify this model
Lefkowich & Richardson (30)	Ireland	Qualitative	NR	Shed members, Ireland	Irish Sheds function as alternative health spaces aligning with masculine norms of doing rather than disclosing; reduce isolation through side-by-side activity
Taylor et al. (23)	Australia	Mixed methods	NR	Shed members, Queensland	Sheds described as ‘home away from home'; improved mood, social connection, sense of purpose, and belonging consistently reported
Foster et al. (22)	Scotland	Mixed methods	NR	Scottish Shed members	Personal and community impact includes reduced isolation, improved wellbeing, and enhanced sense of community contribution
Kelly et al. (29)	UK	Conceptual scoping review	16 studies	Shed members and programmes	Logic model of causal pathways: meaningful activity, routine, social connection, psychological safety, meaning, and identity as key mechanisms to wellbeing
Kelly et al. (21)	Scotland	Mixed methods	NR	Men's Sheds in Scotland	Scottish Sheds improve health and wellbeing; social connection and activity engagement are primary drivers; health by stealth model operational
Kelly & Steiner (24)	Scotland	Quantitative	NR	Shed members	Limited documented impact on physical health outcomes; psychosocial benefits more strongly evidenced; highlights measurement gap
McGrath et al. (19)	Ireland	Mixed methods RCT-adjacent	NR	Sheds for Life participants	Structured health promotion through Sheds improves wellbeing, physical activity, healthy eating, social capital over 12 months; men with lowest baseline show greatest gains
Cavanagh et al. (26)	Australia	Qualitative	NR	Aboriginal men in Sheds	Culturally appropriate Shed activities support Aboriginal men's thriving; cultural safety and elder mentorship are critical; Western model requires adaptation
Foettinger et al. (15)	International	Mixed-methods systematic review	52 studies	Shed members globally	Consistent evidence for improved self-rated health, reduced isolation, and wellbeing especially men 50+. Three success features: appropriate facilities, sufficient funding, participant-driven management
McGrath et al. (36)	Ireland	Cross-sectional survey	NR	Sheds for Life members	Sociodemographic profiling shows members with lower baseline wellbeing show greatest gains; informs targeted health promotion within Sheds
McEvoy et al. (18)	Australia	Quantitative path-model	n=333	Shed members, mean age 70.9	Psychological safety predicts engagement (frequency, duration, diversity); engagement predicts social networks and meaning in life; both predict higher wellbeing and lower loneliness
Clarke et al. (16)	Australia	Quantitative survey	NR	Older Shed members	Social anxiety and depression risk reduced through Shed membership; behavioral activation mediates protective effects on mental health
Clark Banack et al. (17)	Canada	Mixed methods	NR	Rural Alberta Shed members	Canadian rural Sheds support older men's mental health through work-like project-based activity; camaraderie, purpose, and inclusion are primary outcomes
Clarke et al. (20)	Australia	Quantitative survey	NR	Shed members	Identity leadership in Sheds predicts mental health outcomes; shared social identity as a Shed member independently associated with improved wellbeing
Gater et al. (28)	UK/Wales	Qualitative	NR	Self-organized men's mental health groups	Emerging self-organized men's groups parallel Shed model for working-age men; peer support, shared activity, and informal connection drive wellbeing

### 3.1 The men's shed model: origin, structure, and spread

Men's Sheds originated in Australia in the late 1970s as grassroots community organizations providing shared spaces where predominantly older men engage in meaningful practical occupation woodwork, metalwork, repairs, electronics, and gardening while socializing informally (12, 13). Wilson and Cordier's (12) foundational narrative review described the movement as built around a community development philosophy emphasizing ‘learning in community contexts,' with participation voluntary, self-directed, and free from the clinical framing that characterizes formal mental health services. The model spread rapidly from Australia to Ireland, Scotland, the United Kingdom, New Zealand, Canada, and beyond, with national male health policies in both Australia and Ireland explicitly identifying Sheds as ideal locations to address male social isolation and promote health (12, 13).

A defining feature of the Men's Shed model is its ‘health by stealth' ethos: health promotion is embedded within activity and socialization rather than foregrounded as the reason for participation (12, 13). This approach is not incidental but strategically designed. As Wilson and Cordier (12) note, any programme where health promotion is overtly stated as an outcome risks reducing men's engagement, because the very act of seeking help can conflict with masculine self-conception. By positioning the Shed as a place to do things rather than a place to get help, the model sidesteps the role conflict described by Addis and Mahalik (9) and creates an entry point that men who would not attend a mental health service are willing to use.

Foettinger et al. (15) mixed-methods systematic review of 52 studies across multiple countries identifies three consistent features of successful Sheds: appropriate facilities, sufficient and sustainable funding, and participant-driven management and organization. The third feature is particularly important: Sheds where members have significant ownership over programming, activities, and governance consistently outperform those with externally imposed structures in terms of attendance, engagement, and perceived benefit. This finding has direct implications for Canadian implementation, where Sheds remain relatively underdeveloped compared to Australia and Ireland, and where governance models must navigate distinct funding landscapes and demographic contexts (17).

Typological heterogeneity is a persistent challenge in the Shed literature. Sheds vary considerably in their explicit orientation toward health promotion vs. activity provision, in the populations they serve, and in the degree to which health information and professional support are integrated into programming (12, 35). Wilson and Cordier (12) proposed a typology distinguishing utility-focused from socially-focused Sheds, but this classification has not been systematically applied in subsequent research. This heterogeneity complicates evidence synthesis: findings from health-promotion-oriented Sheds with embedded nursing or allied health staff cannot be assumed to generalize to activity-only Sheds, and the absence of consistent typological data across studies means that what 'Men's Sheds' means varies substantially across the literature.

### 3.2 The impact of men's sheds on mental health and wellbeing

#### 3.2.1 Psychosocial outcomes for older and retired men

Foettinger et al. (15) mixed-methods systematic review the most comprehensive synthesis to date, including 35 qualitative, nine quantitative, and eight mixed-methods studies concludes that Shed participation is consistently associated with benefits to self-rated health, reduced social isolation, and improved subjective wellbeing, particularly for men aged 50 and over. While effect sizes are not pooled due to substantial outcome heterogeneity across studies, the convergence of qualitative and quantitative data across multiple countries, contexts, and Shed types represents meaningful cumulative evidence. The review identifies three domains of consistent benefit: psychosocial wellbeing (most strongly evidenced), social inclusion, and health literacy (moderately evidenced), with physical health effects notably weaker and less consistently documented.

The strongest quantitative evidence for the pathway from Shed participation to mental health outcomes comes from McEvoy et al. (18) path-model survey of 333 Shed members in Australia (mean age 70.9 years, 98% male). The structural equation model demonstrated that higher psychological safety feeling accepted, valued, and safe within the Shed environment was associated with greater engagement across three dimensions: frequency of attendance, duration of individual sessions, and diversity of activities participated in. This engagement in turn independently predicted two key intermediate outcomes: larger social networks outside the Shed and greater meaning in life. Both social networks and meaning in life then predicted higher subjective wellbeing and lower loneliness at follow-up, with the overall model explaining a substantial proportion of variance in wellbeing outcomes. Critically, behavioral activation increased engagement in pleasant activities and lower alcohol use were independently associated with wellbeing and health-related quality of life, suggesting that Shed participation may produce benefits through multiple simultaneous pathways rather than a single causal chain.

In Ireland, McGrath et al. (19) evaluation of the Sheds for Life programme used a prospective mixed-methods design with pre- and post-assessments to document improvements in subjective wellbeing, physical activity levels, healthy eating behaviors, and social capital at 12 months following structured health promotion delivered through Sheds. Importantly, men with lower baseline wellbeing, greater loneliness, or who lived alone showed the greatest odds of meaningful improvement, suggesting that Sheds may be particularly valuable for the most socially isolated men precisely the population for whom formal health services have proven least accessible. Clarke et al. (16) further found that social anxiety and depression risk were reduced through Shed membership, with behavioral activation mediating these protective effects, while Clarke et al. (20) demonstrated that identity leadership within Sheds where members develop a strong shared sense of Shed identity and see themselves as embodying what it means to be a Shed member independently predicts improved mental health outcomes above and beyond the effects of general social participation.

Qualitative and mixed-methods evidence from Scotland (21, 22), Australia (23), and rural Alberta (17) consistently corroborates these quantitative findings. Taylor et al. (23) describe Sheds as functioning like a ‘home away from home,' a phrase that captures the qualitative texture of what the quantitative models are measuring: a place where men feel belonging, routine, and social connection in a way that mirrors the social world of employment that retirement disrupts. Foster et al. (22) documented reduced isolation, improved sense of purpose, and enhanced community contribution in a Scottish Shed, while Clark Banack et al. (17) found that members of two rural Alberta Sheds reported increased camaraderie, sense of purpose, and social inclusion, and concluded that Sheds represent a worthwhile investment for supporting vulnerable rural men's mental health, while noting they should not be positioned as substitutes for formal mental health services.

Evidence for physical health impacts is considerably weaker. Kelly and Steiner's (24) quantitative study of Scottish Sheds found limited documented impact on physical health outcomes, and Foettinger et al. (15) systematic review similarly identified physical health as the domain least strongly supported by the evidence base. This pattern is consistent across the literature and suggests that the primary mechanism of benefit operates through psychosocial rather than physiological pathways an important consideration for how Sheds are positioned within broader health promotion frameworks.

#### 3.2.2 Outcomes for diverse and underserved male populations

The evidence base for Men's Shed outcomes is heavily concentrated in older, white, Anglophone men in Australia, Ireland, Scotland, and the United Kingdom. This demographic concentration substantially limits the generalizability of findings and their applicability to diverse Canadian populations. However, the available evidence on underserved groups is instructive.

For Indigenous men, Southcombe et al. (25) found that capacity-building through Indigenous men's groups and Shed-adjacent programs in Australia required culturally adapted approaches that centered elder leadership, connection to land, and community governance rather than the Western workshop model. The study documented improvements in social connection, mental and emotional wellbeing, and community cohesion, but emphasized that these outcomes were contingent on genuine cultural adaptation rather than superficial modification of existing Shed structures. Cavanagh et al. (26) similarly found that Aboriginal men ‘thrived' through culturally appropriate Shed-based activities that aligned with Indigenous masculine identities and community values, with elder mentorship and cultural safety identified as critical success factors. The central implication is that the Men's Shed model is not culturally neutral: its alignment with Western, white masculine norms around woodworking and tool use may represent both a strength for its traditional target population and a barrier for men whose masculine identities are shaped by different cultural frameworks. In the Canadian context, there are no published studies on Men's Sheds specifically serving First Nations, Métis, or Inuit men, representing a critical evidence gap given disproportionate rates of social isolation and mental health burden in these communities.

For racialized and immigrant men, no included studies specifically examined outcomes in these populations within Men's Sheds. Henwood et al. (27) note the presence of culturally and linguistically diverse men in Australian men's health community organizations, but do not report outcomes disaggregated by ethnicity or immigration status. This gap is particularly consequential for Canadian urban contexts Edmonton, Toronto, Vancouver where immigrant men represent a substantial proportion of the older male population and where cultural alignment with the traditional Shed model may be limited. For 2SLGBTQ+ men, no included studies examined experiences or outcomes in Men's Sheds, and the male-only, traditionally masculine environment of most Sheds may present structural barriers to participation for gay, bisexual, transgender, or non-binary men. For working-age men, the evidence base remains almost entirely focused on older and retired men. Gater et al. (28) recent qualitative study of self-organized men's mental health groups in Wales represents an emerging exception, documenting peer support and social connection benefits for working-age men in group settings that parallel the Shed model, and suggesting that the core mechanisms may be transferable across age groups with appropriate programming adaptation.

#### 3.2.3 Methodological quality of outcome evidence

The quality of outcome evidence across the Men's Shed literature is consistently limited by several interrelated problems. First, the predominance of cross-sectional and qualitative designs prevents causal inference: we know that men who participate in Sheds report better outcomes, but cannot determine from most studies whether Shed participation caused these improvements or whether men with better psychosocial resources are more likely to engage and remain engaged. Wilson and Cordier's (12) foundational review noted that most studies were descriptive surveys or small qualitative projects, and Foettinger et al. (15) systematic review confirms that this remains true across the expanded literature. Only McEvoy et al. (18) and McGrath et al. (19) provide quantitative designs with adequate sample sizes to meaningfully test causal pathways, and neither has been replicated.

Second, the absence of standardized outcome measures makes cross-study comparison effectively impossible. Few studies use validated instruments: McEvoy et al. (18) used the Warwick-Edinburgh Mental Wellbeing Scale (WEMWBS) and the UCLA Loneliness Scale among others, while McGrath et al. (19) used the Short Warwick-Edinburgh Mental Wellbeing Scale (SWEMWBS), but the majority of studies rely on bespoke questionnaires, self-report ratings, or qualitative data without standardized anchors. The field would benefit substantially from adoption of a shared core outcome set including the UCLA-3 for loneliness, WEMWBS for wellbeing, PHQ-9 for depression, GAD-7 for anxiety, and CMNI-46 for masculinity norms, which would enable the cross-study synthesis and eventual meta-analysis that the field currently lacks.

Third, demographic reporting is frequently inadequate. Many studies report only age and gender, with limited detail on socioeconomic status, ethnicity, prior mental health history, or rurality, making it impossible to assess for whom Sheds work best and under what conditions (36). Kelly et al. (29) scoping review reports that demographic and contextual details were often sparse, a finding that substantially limits the practical utility of the evidence base for commissioners and policymakers.

### 3.3 Mechanisms: how men's sheds produce mental health benefits

#### 3.3.1 Activity engagement and meaningful occupation

The most consistently identified mechanism across the Men's Shed literature is engagement in meaningful, practical activity. This mechanism operates through several distinct but interrelated pathways that together explain why doing things together rather than simply being together is central to how Sheds work.

The first pathway is the restoration of a sense of usefulness and competence after retirement. For many older men, paid employment has been the primary source not only of income but of identity, social connection, daily structure, and a sense of being needed (12, 23). Retirement disrupts this structure acutely, and the literature consistently documents that men who lack alternative sources of purposeful activity after retirement are at substantially elevated risk of social isolation and mental health deterioration. Wilson and Cordier (12) identified this pattern across their review of foundational Shed studies in Australia, where participants repeatedly described the Shed as filling the void left by work: a place where skills accumulated over a lifetime remain valued and useful, where there is always something to be done, and where the doing carries social meaning because it benefits others community organizations, families, charities. Taylor et al. (23) capture this quality in the phrase ‘home away from home,' which implies not merely comfort but belonging and purpose in a space that feels like one's own.

The second pathway is the provision of routine and structure. Regular Shed attendance typically one or more sessions per week, often on fixed days provides a temporal scaffold that combats the purposelessness and temporal disorientation that isolation and retirement can produce (12, 17, 29). Kelly et al. (29) logic model identifies routine as a distinct causal pathway to wellbeing that operates independently of the social connection produced by attendance: the act of having somewhere to go, a commitment to keep, and a predictable structure to the week has intrinsic psychological value beyond the social content of what happens at the Shed.

The third pathway is activity-based behavioral activation. McEvoy et al. (18) path model found that behavioral activation engagement in rewarding and pleasant activities was independently associated with both subjective wellbeing and health-related quality of life, even after controlling for social network size and meaning in life. This finding situates Shed participation within the established evidence base for behavioral activation as a mechanism for improving mood and reducing depression risk (16, 20), and suggests that the specific character of Shed activities hands-on, productive, visible in their outcomes, and oriented toward community benefit may be particularly well suited to generating the activation effects that less structured social participation does not reliably produce.

The quality of evidence for the activity engagement mechanism is moderate. The pathway is supported by consistent qualitative evidence across multiple countries and contexts (12, 17, 21–23, 30), and receives quantitative support from McEvoy et al. (18) path model in the domains of behavioral activation and engagement diversity. However, no study has directly compared structured activity-based Shed participation with unstructured social participation to isolate the specific contribution of the activity component, and the qualitative evidence base relies primarily on retrospective self-report rather than prospective measurement.

#### 3.3.2 Social connection and peer support

Social connection is the most strongly evidenced mechanism in the Men's Shed literature and the one for which the quantitative evidence is most compelling. McEvoy et al. (18) path model demonstrates that Shed engagement directly predicts larger social networks both within and outside the Shed, and that larger social networks in turn predict higher wellbeing and lower loneliness. This finding is consistent with the extensive epidemiological literature on social isolation reviewed in the Introduction, and establishes a direct causal pathway from Shed participation to the reduction of the very isolation that brings men to Sheds.

The qualitative literature provides important texture around how this social connection is built and why it takes the particular form it does in Shed settings. A consistent theme across studies in Australia (12, 23), Ireland (30), Scotland (21, 22), and Canada (17) is the importance of what has been described as ‘shoulder-to-shoulder' or ‘side-by-side' socialization: conversation that arises naturally from shared activity rather than being the explicit purpose of attendance. Men who would not attend a group convened for the purpose of talking about their feelings will work alongside another man for hours, and in the course of that work share thoughts about their lives, their families, their health, and their struggles not because they have decided to seek help but because the activity creates the relational conditions in which conversation becomes natural and disclosure becomes possible without requiring the acknowledgment of vulnerability that formal help-seeking demands (33). Lefkowich and Richardson (30) describe this dynamic as central to how Irish Sheds function as alternative health spaces: the activity is not merely instrumental to social connection but constitutive of it, creating a shared focus that makes conversation feel incidental even when it is doing significant psychological work.

Psychological safety, as conceptualized and measured by McEvoy et al. (18), is the precondition for this form of social connection. Feeling safe, accepted, and valued within the Shed environment predicts greater engagement across all three dimensions measured frequency, duration, and activity diversity which then predicts the expansion of social networks. This finding has important practical implications: it suggests that the quality of the relational environment within the Shed matters as much as the activities offered, and that Sheds which inadvertently reproduce exclusionary masculine dynamics hierarchies, competitiveness, bullying may undermine the very mechanism through which their benefits are produced. The participant-driven management model identified by Foettinger et al. (15) as a feature of successful Sheds may be important precisely because it supports the psychological safety that engagement and social connection require.

Clarke et al. (20) finding that identity leadership predicts mental health outcomes adds a further dimension to the social connection mechanism. When Shed members develop a strong, shared social identity as members when they think of themselves as 'a Shedder' and feel pride in that identity this shared identity becomes a source of belonging and self-worth that extends beyond the individual sessions and contributes to mental health outcomes that persist across time. This mechanism, grounded in social identity theory, provides a theoretical account of why Sheds may produce durable benefits rather than effects that fade when attendance lapses.

#### 3.3.3 Masculinity norms as facilitator and constraint

Masculinity norms occupy a paradoxical position in the Men's Shed literature: they are simultaneously the primary reason that formal mental health services fail to reach many men, and the primary reason that Men's Sheds succeed in reaching them. Understanding this paradox is essential to understanding both the value and the limitations of the Men's Shed model.

As facilitator, the alignment of Shed programming with hegemonic masculine ideals centered on productivity, competence, practical skill, and tangible contribution lowers the entry barriers that prevent men from accessing support (39). Connell's (8) framework of hegemonic masculinity describes how dominant masculine ideals create a hierarchy within which certain ways of being a man are valorized and others are stigmatized. Formal mental health services which require men to acknowledge vulnerability, seek help from an expert, and discuss emotional experiences in a clinical setting sit at the stigmatized end of this hierarchy for many men, particularly older men whose masculine self-conception was formed in cultural contexts with little tolerance for male emotional expression. Sheds, by contrast, offer an entry point that is congruent with hegemonic masculine identity: a man who goes to a Shed is doing something useful, developing or applying skills, contributing to his community, and socializing with other men all activities consistent with valued masculine identity (12, 13, 30). The help-seeking, if it occurs at all, is incidental and invisible rather than explicit and shameful.

This alignment is not merely passive. The ‘health by stealth' model (12, 13, 30) represents an active design choice to embed health promotion within the activity context in ways that do not require men to identify as patients, clients, or help-seekers. McGrath et al. (19). Sheds for Life programme in Ireland is the most formally developed example: structured health promotion modules covering physical activity, nutrition, social capital, and mental wellbeing were delivered through the Shed environment, achieving outcomes that clinic-based delivery to the same population would be unlikely to match, precisely because the Shed context made engagement possible for men who would not attend a health programme.

As constraint, the same alignment with hegemonic masculinity that makes Sheds accessible to many men may limit their capacity to produce the deeper changes in masculine identity and help-seeking behavior that would constitute genuine gender transformation. Lefkowich and Richardson (30) argue that Irish Sheds function as ‘alternative health spaces' that allow men to discuss health within the norms of masculine activity rather than challenging those norms a distinction that matters for whether Sheds merely reproduce existing masculine patterns or contribute to their renegotiation. Critical men's health scholarship has raised the concern that Sheds may inadvertently reinforce the very norms that produce men's health disadvantage: by validating woodworking and tool use as the natural context for male social life, they may implicitly confirm that emotional disclosure and help-seeking are not masculine activities, and thereby deepen rather than challenge the stigma they seek to circumvent.

Cavanagh et al. (26) study of Aboriginal men adds a further layer of complexity: for men whose masculine identities are not organized around Western norms of individual productivity and technical skill, the traditional Shed model may be culturally misaligned in ways that require not just adaptation but reconceptualization. The finding that Aboriginal men thrived through culturally appropriate Shed activities where the masculine ideals being affirmed were Indigenous rather than Western suggests that the mechanism of masculine-norm alignment is real and powerful, but that the specific norms being aligned with must match the cultural context of the population being served.

Critically, masculinity norms are rarely measured directly in Men's Shed research. Most claims about the relationship between Shed participation and masculine identity are inferred from qualitative descriptions of participants' experiences rather than empirically tested using validated instruments such as the CMNI-46 (31). This leaves the theoretical claim that Sheds negotiate masculine norms as largely inferential, and means that fundamental questions whether Shed participation changes men's conformity to masculine norms over time, and whether Sheds that explicitly aim to challenge masculine norms produce better or worse outcomes than those that simply align with them remain unanswered.

### 3.4 Equity, diversity, and the limits of generalizability

The Men's Shed evidence base reflects a significant demographic narrowness that limits both its scientific generalizability and its applicability to diverse Canadian communities. The literature is dominated by studies of older, white, Anglophone, cisgender, heterosexual men in Australia, Ireland, Scotland, and the United Kingdom. This concentration is not merely a sampling limitation but reflects a structural feature of the Men's Shed movement itself: Sheds originated in and were designed for a specific demographic, and their expansion has occurred primarily within culturally similar populations.

For Indigenous men in Canada, there are no published studies on Men's Sheds specifically serving First Nations, Métis, or Inuit populations. The Australian evidence from Southcombe et al. (25) and Cavanagh et al. (26) is instructive but not directly transferable: the cultural, historical, and political contexts of Indigenous men in Canada differ substantially from those in Australia, and the adaptation required to serve Canadian Indigenous men would need to center Indigenous epistemologies, governance structures, and masculine identities in ways that go beyond the models currently documented in the literature. For racialized and immigrant men, the evidence gap is equally complete within the Shed literature, and particularly consequential given that immigrant men in Canadian cities face compounding risks of isolation language barriers, unfamiliarity with health systems, disrupted social networks, and discrimination that the traditional Shed model is not designed to address. For 2SLGBTQ+ men, the assumption of cisgender heterosexuality embedded in virtually all Shed research and programming represents not merely an evidence gap but a potential structural barrier, and no included study has examined whether the traditional Shed environment is welcoming or hostile to gender and sexually diverse men. For working-age men, the evidence base provides almost no guidance, despite the well-documented social isolation burden among unemployed, single, and recently separated men under 50 (1, 5). Gater et al. (28) emerging evidence on self-organized men's mental health groups in Wales suggests that Shed-adjacent models can produce benefits for working-age men, but this work is preliminary and not yet replicated.

These gaps are not merely academic concerns. Canada's commitment to equity in public health requires that community-based mental health programs be designed to reach the men who bear the greatest burden of isolation and mental health disadvantage and the current evidence provides insufficient guidance for reaching Indigenous, racialized, immigrant, 2SLGBTQ+, or working-age men through Shed-based programming. Future research must prioritize these populations, and Canadian Shed development must be accompanied by community-based participatory research that centers the perspectives and needs of diverse male communities in program design and evaluation (34, 37, 38).

## 4 Discussion

This narrative review synthesizes evidence on three mechanisms activity engagement, social connection, and masculinity norms through which Men's Sheds appear to produce mental health benefits for older and retired men. The evidence, while heterogeneous in quality and limited in its demographic scope, converges on a coherent picture: Sheds work because they offer men a way to access belonging, purpose, and peer support through activity that is congruent with masculine identity rather than requiring the acknowledgment of vulnerability that formal mental health services demand. This is a genuinely important contribution to public mental health, and one that is particularly relevant to Canada, where male social isolation is well-documented (4, 6), men's underutilization of formal services is persistent (3, 10, 32), and community-based alternatives remain underdeveloped.

The three mechanisms reviewed are interconnected and mutually reinforcing in ways that the literature has only partially characterized. Activity engagement produces routine and behavioral activation, which support mood and wellbeing directly while also creating the relational conditions for social connection. Social connection, when it develops within a psychologically safe environment, produces expanded networks, meaning in life, and identity affirmation, which produce durable improvements in loneliness and wellbeing. Masculinity norms both shape the conditions under which the first two mechanisms operate making activity-based, side-by-side socialization more accessible than clinic-based help-seeking and constrain their transformative potential, by limiting the depth of emotional disclosure and help-seeking that Sheds can facilitate for men who remain tightly bound to hegemonic masculine identities. Understanding how these three mechanisms interact, and for which men and under which conditions each mechanism is most important, is the central theoretical and empirical task for the next generation of Men's Shed research.

Viewed through Connell's hegemonic masculinity framework (8) and Addis and Mahalik's help-seeking model (9), the Men's Shed model represents a pragmatic accommodation to the reality of masculine identity rather than a challenge to it. Men who would not present to a mental health service will attend a Shed to do woodwork and in doing so access peer support, social connection, and identity affirmation. Whether this accommodation constitutes genuine progress toward gender-transformative health promotion or simply a more effective way of delivering support within existing masculine norms is a theoretically and politically important question that the current evidence cannot resolve. What the evidence does suggest is that the answer may differ across contexts: the Sheds that produce the deepest benefits for the most isolated men may be precisely those that challenge, rather than merely accommodate, the norms that produced that isolation in the first place.

The limitations of the current evidence base are substantial and must inform how Shed outcomes are communicated and how programs are funded and commissioned. The predominance of cross-sectional and qualitative designs means that causal claims must be made cautiously; the absence of standardized outcome measures means that effect sizes cannot be estimated and comparisons across contexts are unreliable; and the demographic narrowness of the research means that evidence-based guidance for diverse populations is largely unavailable. These are not arguments against Sheds the convergent evidence from multiple contexts and designs is meaningful but they are arguments for a much more rigorous research agenda that employs longitudinal designs, validated instruments, and community-based participatory methods to build the evidence base that the policy and practice momentum of the Men's Shed movement now requires.

### 4.1 Positionality

The author is the founder of a Men's Shed in Edmonton, Alberta, Canada, established in 2023. This role provides contextual familiarity with Shed programming, the challenges of community implementation, and the social dynamics of male-focused group settings in a Canadian urban context. However, it also represents a potential source of confirmation bias in the interpretation of the literature, as the author has a personal and community investment in the Men's Shed model. To mitigate this risk, findings are presented with explicit attention to methodological limitations and contradictory evidence, claims are restricted to what the peer-reviewed literature supports, and no observations from the Edmonton Shed are presented as evidence in this review.

## 5 Conclusion

Men's Sheds have emerged as a meaningful and practically important community-based response to male social isolation and mental health needs, particularly among older and retired men. The evidence synthesized in this review supports the view that Sheds promote mental wellbeing primarily through three intersecting mechanisms: engagement in meaningful practical activity that restores purpose, competence, and routine; peer social connection fostered through side-by-side activity in a psychologically safe environment; and alignment with masculine norms that reduces the role conflict and stigma that prevent many men from accessing formal mental health services (12, 13, 15, 17, 18, 29, 30).

Yet the evidence base remains developing, and important questions remain unanswered. To establish Men's Sheds as evidence-based mental health interventions capable of guiding policy and practice, future research should: (14) employ longitudinal and, where feasible, experimental or quasi-experimental designs that support causal inference; (15) adopt standardized outcome instruments including the UCLA-3 for loneliness, WEMWBS for wellbeing, PHQ-9 for depression, GAD-7 for anxiety, and CMNI-46 for masculinity norms; (13) incorporate explicit assessment of how Shed participation affects conformity to masculine norms over time, to determine whether Sheds reproduce, renegotiate, or recompose hegemonic masculinity; (16) prioritize equity by examining outcomes for Indigenous, racialized, immigrant, 2SLGBTQ+, and working-age men in diverse national contexts; and (28) integrate Shed models with broader social prescribing and primary care strategies, particularly in Canadian health systems where community-based mental health infrastructure remains underdeveloped relative to need. The social significance of Men's Sheds as a model for reaching men who would not otherwise access support is substantial; the scientific and policy work required to fulfill that potential is the task now before the field.
